# Process Factors in Long-Fiber Thermoplastic Compression Molding Materials

**DOI:** 10.3390/polym18070806

**Published:** 2026-03-26

**Authors:** Christoph Schelleis, Andrew Hrymak, Frank Henning

**Affiliations:** 1 Fraunhofer Institute for Chemical Technology ICT, Joseph-von-Fraunhofer-Str. 7, 76327 Pfinztal, Germany; 2 Institute of Vehicle Systems Technology, Karlsruher Institute of Technology, Rintheimer Querallee 2, 76131 Karlsruhe, Germany; 3 Chemical and Biochemical Engineering, Western University, 1151 Richmond St. N., London, ON N6A 3K7, Canada; ahrymak@uwo.ca

**Keywords:** LFT-D, parameter optimization, overview, doe study, response contour plot, microstructure, composite

## Abstract

Long-fiber thermoplastic (LFT) materials are a versatile category of composite materials that can be directly compounded (LFT-D) in twin screw extruders and compression molded. Originating in the automotive sector, the LFT-D process is becoming increasingly attractive for other industries where low cycle times, lightweight performance and recyclability are required. The purpose of this work is to summarize mechanical properties and findings from the investigations into LFT-D process–microstructure–property relationships and present a design of experiments (DoE) study based on the current state of the art. Primary parameters from LFT-D compounding, screw speed, fiber roving amount and polymer throughput *m*_p_ are chosen as DoE factors. Polyamide 6 (PA6) is reinforced with a glass fiber (GF) mass fraction *w*_f_ between *w*_f_ = 20% and *w*_f_ = 60%. Tensile, flexural and impact properties are chosen as DoE output parameters, characterized and discussed in relation to the state of the art. The unique microstructure of LFT-D materials, especially the existence of a charge and flow area as well as the fiber migration, is considered in the discussion. All mechanical properties characterized have a linear relation to *w*_f_. This study demonstrates the interactive relationship between the main factors and *w*_f_, which significantly influences the mechanical properties. This dependence of *w*_f_ on the DoE factors is accounted for in advanced response contour plots proposed in this work. Parameter recommendations for the screw speed are reported by ranges of *w*_f_ and polymer throughput for the goal of maximum mechanical properties or low coefficient of variations. At *w*_f_ < 30% a low screw speed is recommended to improve most mechanical properties as well as the coefficient of variation.

## 1. Introduction

Composites, the combination of reinforcing fibers and a polymer matrix, are an established but still innovative material class widely used in engineering applications from aeronautical to automotive applications, from electronics to infrastructure [1]. Lightweighting of parts directly correlates with a better fuel economy, especially crucial in flight applications where this translates into longer ranges, longer flying time or higher payload. Composite manufacturing in aerospace applications has been semi-manual for decades, with common processing steps being layup of fiber plies or pre-preg systems, compacting, curing under temperature and pressure [2]. With growing demands in electrified air mobility for passenger transport, goods delivery or military application, the productions of manned and unmanned aerial vehicles are projected to grow. Mass production of quality composite parts at low costs per part is well known to the automotive industry, where composites are also widely used. The dominant processes here are injection and compression molding [3]. These processes work very well with thermoplastic polymers featuring an inherent recyclability and weldability, as opposed to thermoset resin types typically used in aerospace manufacturing to date. Composites can also be categorized by the matrix polymer, i.e., thermoset or thermoplastic. While the market is split by matrix polymer, thermoplastic materials gain ground every year, reaching 60% market penetration in 2022. Thermoplastic polymer systems are overwhelmingly processed in injection and compression molding, with injection molding being the more common method at 25% market share by volume for thermoplastic polymer systems [3,4].

### 1.1. Motivation, Approach and Goals

Processes and materials used today in the automotive sector could deliver the quantities needed in certain aerospace applications of sufficient quality. Long-fiber thermoplastic (LFT) materials compounded directly (LFT-D) and processed in compression molding are a versatile class of composites. The choice of manufacturing route and material system needs to be backed by reliable data. Material property references as well as structured investigations into the process–microstructure–property relationships are lacking in the state-of-the-art processes.

In the following literature review, we are showcasing the mechanical properties most important for LFT-D materials falling within a broad range and not collected in a standardized manner until now. We also strive to present a comprehensive overview of the latest progress regarding process factor influences and suggest a methodological design of experiments (DoE) approach to investigate the process–microstructure–property relation in LFT-D compression molding regarding the main factors identified. Experiments rarely consider the close connection between the main factors and the fiber content. The manufacturing process is divided into two phases. While LFT-D compounding is continuous, the compression molding step is discontinuous. The effects of the extrusion parameters cannot be directly linked to the mechanical properties from a part. We present an advanced response contour plot that combines processing factors, the fiber content and mechanical properties. The goal is to give screw speed setting recommendations for high mechanical properties, or a low coefficient of variation based upon the contour plots introduced. Parts of this work were presented in the doctoral thesis of Schelleis and are identified accordingly [5].

### 1.2. Brief Introduction to Fiber-Reinforced Polymers

A fiber-reinforced polymer (FRP), is a synergy between very different components that form a product better than the individual component [6]. Mechanical loads are transferred from matrix to fiber. The relevant fiber properties are fiber volume content *v*_f_, fiber length *l*_f_ and fiber orientation φ_f_ [6,7]. During processing and the rest of this work, however, the fiber weight content *w*_f_ is used. This key property *w*_f_ is the ratio of fiber mass *m*_f_ to total mass *m*_LFT_ = *m*_f_ + *m*_p_ shown in Equation (1) [6].

*w*_f_ = *m*_f_ × (*m*_f_ + *m*_p_)^−1^, (1)

where *m*_LFT_ comprises *m*_f_ and polymer mass *m*_p_.

Fiber length in DiCo FRP can be set for sheet molding compound (SMC) or glass mat thermoplast (GMT) but has a fiber length distribution (FLD) for most other directly processed materials. Mechanical properties will, in theory, steadily increase with *l*_f_. However, longer fibers tend to bundle and eventually decrease performance making a target *l*_f_ an important optimization goal [8]. Thomason determined experimentally the general connection between mechanical properties and *w*_f_ and *l*_f_, finding that the stiffness reaches saturation first, strength follows at higher *l*_f_ and impact properties only benefit from long fibers [9,10,11,12,13]. The *l*_f_ in LFT-D is not uniform but the FLD shows shorter fibers due to fiber attrition during processing, which breaks down the mean *l*_f_. What constitutes a long fiber is debatable, with demarcations at *l*_f_ = 1 mm [14], at *l*_f_ = 5 mm [15], from at *l*_f_ = 3 mm to *l*_f_ = 25 mm [16] or as high as *l*_f_ = 50 mm [17]. The production process is disregarded in these statements, as fiber lengths of 1 mm are certainly high for an injection molding part but low for a compression molding part [18]. The weight-averaged fiber length *l*_w_ was found to better represent the intensity of longer fibers in mechanical property predictions [19].

Orientation measurements of thousands of individual fibers can be displayed as a histogram, which is the discrete approximation of the fiber orientation distribution (FOD) function ψ_φ_ (φ) [7], where the fiber orientation is the vertex of ψ_φ_ at the angle φ_f_.

### 1.3. Processing LFT-D in Compression Molding

**Figure 1:**
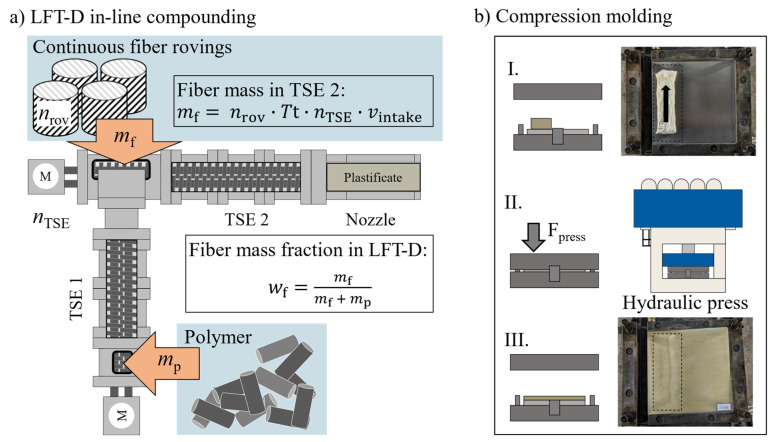
LFT-D processing scheme [24]. (**a**) LFT-D in-line compounding, factors and governing equations. (**b**) Compression molding steps: (**I**) charging the mold with the plastificate; (**II**) closing and filling the mold; (**III**) de-molding the part with resulting charge and flow area.

Good mechanical performance, decent moldability and short cycle times place the LFT compression molding process between injection molding and GMTs [15,18,20,21]. Different machine concepts for melting and mixing such as twin screw extruders (TSEs) machines are common [22,23]. In the single extruder process route, both polymer compounding as well as fiber incorporation are done on the same screw set with the same screw speed *n*_TSE_. The LFT-D process features a couple of distinct characteristics regarding processing parameter dependencies and resulting microstructure generation that will be discussed here. Two TSEs arranged in a cascade form the core of an LFT-D line shown schematically in Figure 1.

The first TSE1 does the polymer compounding, and a melt film is transferred into the second TSE2. With this melt film, the continuous fiber rovings are drawn into the second TSE, impregnated, cut and dispersed. Extrusion is dominated by the viscous energy dissipation of the polymer when changing from solid to fluid during processing. Rheological properties under pressure and shear force are important [25]. Fibers degrade during the interaction of the fiber with other fibers, the polymer or the machine (i.e., barrel wall or screw) [23]. Fibers still present in bundled form from the roving are protected and conserve *l*_f_ until the bundle is dispersed [8].

Once the fibers are impregnated with the matrix at the end of TSE2, the plastificate is formed by a nozzle [26]. It is a mass of continuously extruded material that is portioned by cutting and placed in the open mold for forming (Figure 1b(I)) [18]. This placement area is also called the charge (C) area. The fibers in the plastificate core are oriented in a double-helix shape [27], which is important for resulting fiber orientations [28]. On the outside, a thin fiber layer is oriented in the extrusion direction indicated by a black arrow in (Figure 1b(I)). The plastificate is known to swell [29], also called lofting, meaning it increases in volume by the restoring force of fibers contained [30]. This lofting ends as soon as the mold closes and material is pushed into the remainder of the mold cavity, forming the flow (F) area (Figure 1b(II)). Once the material has cooled down to mold temperature, it is demolded. This time is dependent on processing temperatures and part thickness but is usually within the timeframe of around a minute. This time dependency highlights the segregated nature of both processing phases where the plastificate is the link between LFT-D compounding and compression molding. No direct link can be established between the processing factors and mechanical properties.

**Figure 2:**
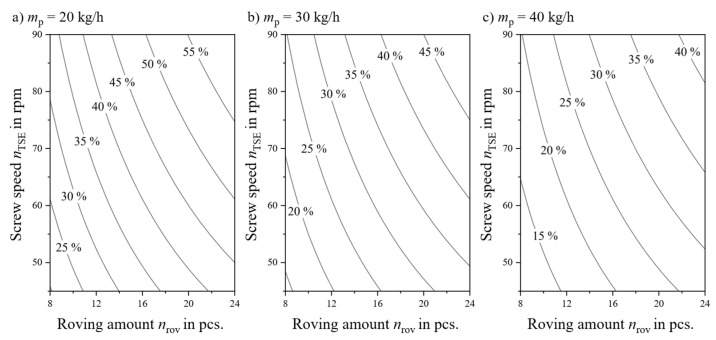
Parameter spaces and resulting *w*_f_ of factors *n*_TSE_ and *n*_rov_ for different *m*_p_. Based on [24].

When processing fiber-reinforced polymers, the fiber fraction *w*_f_ is one of the central factors. The *w*_f_ cannot be directly set on an LFT-D line, but rather is the product of the polymer mass *m*_p_ and fiber mass *m*_f_ (cf. Equation (1)). The *m*_p_ can be set through gravimetric dosing at the LFT-D line. The *m*_f_ is set though *n*_TSE_ and *n*_rov_. As the fibers are wound around the screw, a certain length of fiber is dragged into the extruder per revolution. This length is converted into a weight via the linear density in tex *Tt*. This is reflected in the following Equation (2) [24]:

*m*_f_ = *n*_rov_ × *Tt* × *n*_TSE_ × *v*_intake_, (2)

where a special factor *v*_intake_ is introduced to account for the screw geometry. It describes the length of roving drawn into the extruder per revolution and is dependent on *n*_TSE_ and even the position of the roving relative to the intake slot. An averaged *v*_intake_ value is sufficient to predict *w*_f_. From (2) a set of curves can be derived and plotted, which is shown in Figure 2 [5]. When processing continuous fiber rovings, *w*_f_ will change when a single factor, here *m*_p_, *n*_rov_, or *n*_TSE_, is adjusted [31]. For proper comparability of factor influences, the *w*_f_ must remain the same or the experiment must be set up with utmost care to consider the connection between the factors and *w*_f_.

### 1.4. Key LFT-D Extrusion Parameters

The quality of the LFT-D part depends on the microstructure created during processing [6]. The focus here will be on the reported impact of the principal factors *n*_TSE_, *n*_rov_ and *m*_p_. Another often reported influence can be the screw design, especially the amount and position of the mixing elements used in TSE2. Key extrusion characteristics are the throughput ratio $\dot{V}$* and the specific mechanical energy *SME* [25]. The ratio $\dot{V}$* is calculated in Equation (3) from the material volume ${\dot{V}}_{\mathrm{mat}}$ in the extruder, the housing diameter *D* of the extruder and the screw speed *n*_TSE_.

(3) $${\dot{V}}^{*}=\frac{{\dot{V}}_{mat}}{n_{TSE}\;\cdot D^{3}}$$

In ${\dot{V}}_{\mathrm{mat}}$, other primary LFT-D factors *m*_p_ and *n*_rov_ can be found via *w*_f_. This is closely related to the choice of parameters. Generally, the dispersion of fiber bundles will benefit from a lower $\dot{V}$*. At the same time fiber–polymer interactions are increasing with higher shear conditions that are proportional to a rising *n*_TSE_. Hirata et al. introduced $\dot{Q}$* shown in Equation (4), the ratio of total throughput *ṁ*_LFT_ in kg per hour and *n*_TSE_ in min^−1^ simplifying $\dot{V}$* by omitting the material densities and extruder housing diameter [32].

(4) $$\dot{Q}=\;\frac{{\dot{m}}_{LFT}}{n_{TSE}}.$$

The *SME* is a measure of energy input into the material leading to fiber dispersion and breakage [25]. Most of this energy is introduced by *n*_TSE_ and torque associated with *n*_TSE_. Use of the *SME* as a measure for fiber attrition was proposed by Inceoglu et al. [14], with the direct connection between fiber dispersion and *n*_TSE_ remarked upon by Stratiychuk-Dear et al. [33]. Other studies focus on the *SME* as a measure for the advancement of cross-linking in thermosetting polymers [34,35]. The *SME* can be calculated by Equation (5) with the torque at the TSE motor *M*_TSE_ and the total mass flow rate *m*_total_.

(5) $$SME=\frac{2\cdot \pi \cdot n_{TSE}\cdot M_{TSE}\;}{m_{total}}.$$

Regarding the choice of processing parameters in LFT-D compounding for compression molding, little specific advice is given, as parameter interactions are complex [23]. A simple and commonly used approach was formulated by Tröster [36] to set the polymer throughput, roughly matching tool size and processing times:

- Lower screw speed until TSE2 starts to overflow
- Increase screw speed *n*_TSE_ gradually
- Calculate *w*_f_ via Equation (2) and add rovings *n*_rov_ accordingly
- Iterate *n*_TSE_ in case of TSE2 overflow

This state-of-the-art-approach indirectly accounts for different TSE sizes as scaling effects in extrusion do prohibit a direct transfer of a parameter set between extruders of different sizes [25]. This approach is based on minimizing fiber attrition caused by high *n*_TSE_ and is backed by experiments with cut fibers [5,7]. However, some sources consider the feed rate as having a bigger impact on *l*_f_, as the total number of fiber–machine interactions are tied to TSE residence times, which decrease when the feed rate is higher [25,37]. A blanket statement regarding recommended *n*_TSE_ is, however, not permitted when processing direct rovings, as the extrusion parameters are linked as described in Figure 2 and Equation (3).

The following tables provide an overview of reported parameter optimization studies. The results presented are focused on work with a Dieffenbacher LFT-D line available at either Fraunhofer ICT, Pfinztal, Germany and Fraunhofer Innovation Platform for Composites Research at Western University (FIP-Composites@Western), London, Ontario [5].

### 1.5. Reported Influences of Screw Speed

The central importance of *n*_TSE_ was introduced in the previous sections. It is one of the core experimental factors next to the screw design. Generally in extrusion, *n*_TSE_ has an influence on residence times [38], temperatures [30] and shear rates [14,32]. Apart from those general influences Table 1 focuses on reported effects of *n*_TSE_ on *l*_f_ and mechanical properties. The effects are observed in experiments where *n*_TSE_ is either set to a high (low) level or at least higher (lower) than other set points in that same experiment. The statements are therefore made based on these comparisons. While the connection between *n*_TSE_ and *l*_f_ is made, improvements of *l*_f_ are rarely reflected in the mechanical properties.

### 1.6. Reported Influences of Screw Configuration

The screw configuration in TSE2 can be modified in detail, as single elements can be as short as 10 mm depending on the screw diameter and thus it is complicated to compare single designs. Table 2 provides a broad generalization between low- and high-shear configurations to avoid this fragmentation. High shear elements deployed are either kneading blocks or GFM mixing elements (hedgehog elements), which promote distributive mixing [25]. Most literature sources dealing with low- to medium-viscosity polymers agree that at least one GFM is needed to disperse fibers. This dispersion is beneficial for the mechanical properties. For a highly viscous matrix system, like PC, no positive effect of added shear in the screw design was reported, as bundle dispersion was not an issue [41]. While most sources report a decreased *l*_f_ with increased *n*_TSE_ as expected, this is not detrimental to the mechanical properties. This is representative of the complexity of the optimization task that is sometimes overlooked.

### 1.7. Reported Influences of Total Throughput

All energy put into the system over time, be it through *n*_TSE_ or heating, is distributed over the mass conveyed *m*_LFT_. Figure 2 demonstrates that *m*_LFT_, interchangeable with *w*_f_, is always integrally linked to the primary processing factors. While $\dot{V}$* and the *SME*, both closely related to *m*_LFT_, are identified as essential processing parameters in extrusion, they rarely are considered in LFT-D compounding. Table 3 has thus only non-LFT-D sources.

### 1.8. Further Reported Influences on LFT-D Products

The number of fiber rovings is only relevant to processes with direct roving intake. It is usually set after *m*_p_ and *n*_TSE_ to match the desired *w*_f_ [22,36]. In direct fiber feed injection molding, increasing *n*_rov_ led to higher *l*_w_ [23] as well as lower *w*_f_ deviations [31].

No LFT-D experiments were reported where TSE temperatures were investigated. Most of the temperature increase in the TSE is from shear stresses (viscous dissipation) caused by *n*_TSE_ [25]. While *n*_TSE_ does increase the fiber attrition, a higher temperature and consequently lower material viscosity can compensate for that [44].

Fiber bundles do conserve *l*_f_ by supporting themselves against processing influences [23] and are resistant to fiber-polymer effects like buckling [22]. It is highly dependent on the specific machine setup and shear conditions where a bundle disperses [45]. At that moment, *l*_f_ is high and the aspect ratio is low; these conditions are the ideal solution to the optimization challenge between dispersion and retention of *l*_f_.

### 1.9. Fiber Microstructure in LFT-D Compression-Molded Parts

The mechanical properties are defined by the fiber microstructure, which is in turn defined during processing [6,10]. As properties are clearly linked to *w*_f_, the fiber-migration phenomenon needs to be addressed [46]. Fibers do agglomerate towards the end of the flow path in the mold, leading to a difference in *w*_f_ of up to 16% depending on *w*_f_, polymer and fiber type [47] as well as processing parameters [7]. Fiber content is usually determined via thermogravimetric analysis (TGA) where the polymer matrix is burned under controlled conditions, and the weight change of the sample is measured [26]. Other methods like computer tomography (CT) image analysis [48] or a density-based approach [47] are reported. The *w*_f_ is calculated via Equation (1).

While the mechanical properties remain almost unchanged at small fiber orientation angles (+11.25°), there is already a significant drop at somewhat larger angles (+22.5°). Across the literature it is observed that the fiber orientation in the flow area does not match the perceived flow direction [49,50]. This was shown to originate in a skewed distribution of mass in the LFT-D plastificate [51]. For a detailed discussion of the influence of sample orientation as well as sample placement, the work of Scheuring is recommended [52,53]. Fiber orientations can also be determined by analyzing CT images [48,54,55] or by testing tensile discs [36,56].

Aside from the fiber orientation deviations in the F area, the fiber orientation in the C area shows a pronounced shell-core effect [57]. In this area, the fibers on the outside of the plastificate freeze are oriented in the extrusion direction, while those in the core are oriented in the, usually perpendicular, flow direction [24,55]. The C area is sharply defined and needs to be considered separately, as resulting microstructures are complex with poor repeatability [49,58,59].

Fiber length measurements have proven to be complicated for high lengths achieved during LFT-D compounding [49,56]. A comprehensive overview of methods was presented by Goris et al. [60]. The fibers are separated from the matrix by chemical means or incineration and fiber lengths are analyzed by software such as FASEP^®^ [61]. Reported *l*_f_ are shown in Table 4. The *l*_n_ is the arithmetic mean of the *l*_f_ distribution and *l*_w_ is the weight-averaged *l*_f_ [6]. The consideration of the longer fibers is important, as experiments have shown *l*_n_ to be a poor basis for calculating mechanical properties [14].

### 1.10. Mechanical Properties of LFT-D Materials

While a wide range of LFT-D material combinations is reported, the use-case is almost always the transport industry [4]. Accordingly, most data are from the most prevalent polymers PP and PA6 in combination with glass fibers (GFs) or carbon fibers (CFs), the latter being rather exotic in actual applications due to the high price [3]. More specialized polymers like PC [24,40] and PA66 [39,64,65,66] can be processed and have been thoroughly characterized. The mechanical properties for SAN, PET and ABS originate from screening trials and have not been examined in detail [67,68].

Table 5 and Table 6 present a collection of all reported LFT-D material data to date, with mechanical data rounded to whole numbers for ease of comparison. Quasi-static testing is conducted to or in adherence to either ASTM or ISO normative standards. The exact procedure as well as the conditioning of the samples has been omitted here for brevity’s sake, while the sources provide varying degrees of detail. For a detailed study into environmental effects like temperature or humidity on PA6, the work of Scheuring is especially recommended [52]. During data curation, the aim was to choose dried specimens from the F area in 0° direction. Other sample areas and sampling directions are available in literature; however, the comparability is rapidly declining due to the poor availability of sources and different sampling approaches.

Carbon-fiber-reinforced materials are presented in Table 6. Results available are exclusively for PA6 and PA66 composites and no impact properties are reported.

Reported properties are very heterogeneous even at similar *w*_f_ for similar material systems. This originates from an inherent heterogeneity of the fiber microstructure as well as a high sensitivity to sampling location, which is not always considered. The sample geometry does vary. Fiber migration and orientation deviations should be mentioned here.

### 1.11. Simulative Approaches

The initial plastificate fiber orientation is approximated [28,74,75] and can be used to improve mold filling simulations [76,77]. Rheological properties of LFT-D and related materials can be modelled after characterization during mold filling [78,79]. The complex microstructure of LFT-D require a high number of physical samples or a reliable way to generate them in artificial-intelligence-based methods [80]. Other approaches to microstructure generation feature a shaker-algorithm [81,82]. The mechanical properties depending on fiber orientation and *w*_f_ can be predicted using standard models [83].

## 2. Materials and Methods

The experiment is conducted with glass-fiber-reinforced PA6. LFT-D is compounded and compression-molded into plates for mechanical characterization. During compounding, the processing parameters of the LFT-D line are varied according to a DoE scheme.

### 2.1. Materials

For the LFT-D matrix, a PA6, STABAMID PA6 S22 with a masterbatch, was provided by DOMO Engineering Plastics Europe S.p.A., Leuna, Germany. Glass fiber rovings StarRov 895 2400 tex with a PA6 compatible sizing were provided by Johns Manville GmbH, Wertheim, Germany.

### 2.2. Machinery, Processing Parameters and Factors

**Figure 3:**
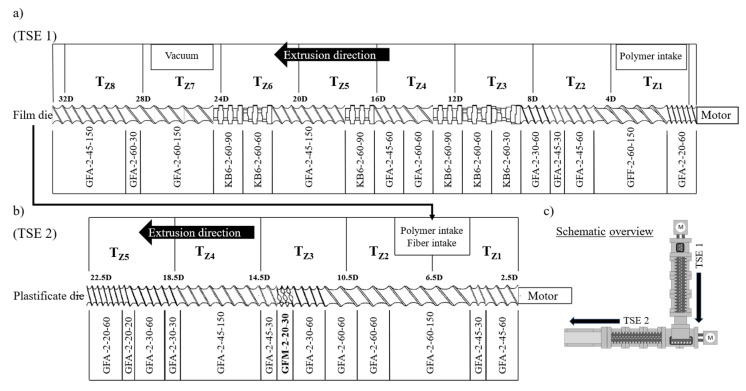
Screw design schematics of TSE1 (**a**) and TSE2 (**b**) [5]. Machine overview (**c**).

LFT-D processing was conducted on an LFT-D ILC line by Dieffenbacher GmbH Maschinen- und Anlagenbau, Eppingen, Germany. It comprises two TSEs by Leistritz AG, Nürnberg, Germany. TSE1 was a Leistritz ZSE 40HP GL/32D with 55 kW nominal power and a barrel diameter of 40 mm. TSE2 was a Leistritz ZSE 40 GL/14.5D with nominal power of 27 kW and a barrel diameter of 40 mm. The screw setups of both extruders are given in Figure 3. Both TSE are arranged perpendicular to each other with TSE1 feeding into TSE2 in an open waterfall die. The fiber intake section of the second TSE is a custom design with a higher inner diameter to allow the fibers to be wound around the screw before being sheared off (temperature zones T_Z1_ and T_Z2_ in Figure 3b). The first pair of screws in TSE1 is a standard all-purpose compounding design. The second pair in TSE2 is its own design with one mixing element (GFM in T_Z3_) for dispersion of fiber bundles. The design was derived from a benchmark of eight screw designs compounding PA6 LFT-D materials. While the screw design is a factor in some experiments in literature, it is not modified during this study.

All T_Z_ of both TSE are set to 275 °C, while the plastificate die at the end of TSE2 is set to 265 °C. The die has a width of 75 mm and is set to a height of 39 mm. The primary parameters are factors in a DoE scheme and described in Section 2.3 Methods.

Compression molding is done on a Dieffenbacher DYL 630/500 parallel-guided hydraulic press by Dieffenbacher GmbH Maschinen- und Anlagenbau, Eppingen, Germany. The closing profile given in Table 7 comprises the gap width *w*_g_ and closing speed *v*_c_. The support points shown are connected in a linear fashion. The press force is set at 3200 kN, representing an in-mold pressure of 200 bar [84]. The plastificate is inserted at the side of the mold as shown in Figure 1b to produce a pronounced flow induced fiber orientation. A distance of 50 mm is always kept between the mold wall and closest plastificate edge. The plastificate is approximately 100 mm wide and will expand its outer layer during early stages of molding. The C area occupies slightly less than half of the form which is considered during sampling.

### 2.3. Methods

For the creation and evaluation of the DoE series the software MODDE 13 by sartorius is used. The system under investigation is the LFT-D line and the hydraulic press with their respective parameters. The experimental design chosen was a Face-Centered-Central-Composite-Design to be able to predict quadratic relationships between the factors. The factors chosen are the principal parameters from LFT-D:

- Screw speed of TSE2 *n*_TSE_ in rpm from 45 rpm to 90 rpm.
- Polymer throughput *m*_p_ in kg/h from 20 kg/h to 40 kg/h.
- Number of rovings *n*_rov_ in pieces from 8 pcs. to 24 pcs.

A trial plan is derived from the low, medium and high set points of each parameter. Those set points reflect at least one limit of the TSE2 and were chosen to be as broad as possible. Table 8 exhibits parameter combinations along with the expected *w*_f_ calculated by Equation (2). The trials were conducted in randomized order as displayed in Table 8. The combinations of parameters result in a *w*_f_ spanning from 19.72% to 59.82%, not yet considering fiber-migration in the mold. Output parameters are the mechanical properties.

### 2.4. Microstructural Characterization

**Figure 4:**
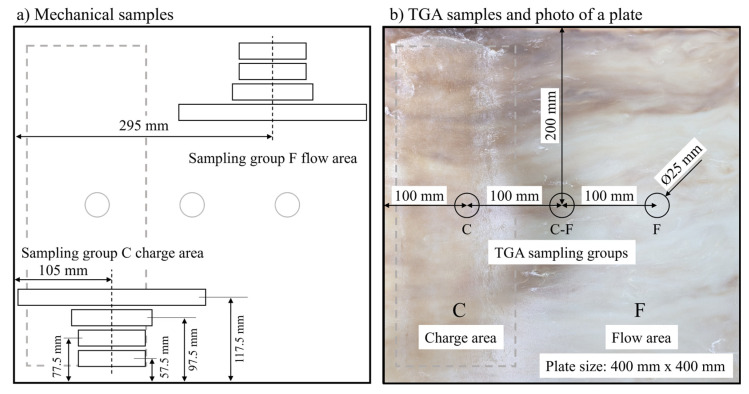
Sampling schemes for (**a**) mechanical testing and (**b**) TGA overlayed on a photo of a plate with C area (dashed rectangle) and F area visible [5].

The TGA is performed on three locations of every plate indicated in Figure 4b. Samples are heated at a rate of 10 °C per minute and burned off at 650 °C for 120 min in a TGA801 by LECO Instrumente GmbH, Mönchengladbach, Germany. The local *w*_f_ is attributed to the sample areas in charge C and flow F.

Fiber lengths are measured with FASEP^®^ after subsampling the TGA samples to contain between 5.000 and 10.000 fibers per measurement. The samples are diluted twice in water and are stirred gently in an ultrasonic bath to separate bundles without damaging the fibers [85].

### 2.5. Mechanical Characterization

Samples are cut from the C and F area according to the scheme in Figure 4a. Six samples per DoE set point are tested for every mechanical property in the C as well as F area. The position of the plastificate is indicated by a grey dashed rectangle. The plastificate position is visible on the plates as the material freezes onto the mold upon contact.

Samples were dried at 80 °C according to DIN EN ISO 1110 before characterization [86]. All samples were 3 mm thick with deviations of ±0.1 mm to be expected. Tensile tests were done in reference to DIN EN ISO 527-1 [87] on samples with a length of 200 mm and a width of 15 mm. Flexural properties were determined according to DIN EN ISO 14125 [88], with 80 mm by 15 mm samples. Charpy impact properties were determined in accordance with DIN EN ISO 179-1fU [89] on 75 mm by 15 mm samples.

## 3. Results and Discussion

The results are presented in ascending order of complexity. Fiber migration is important for the correct display and discussion of mechanical properties. The mechanical properties are shown in relation to their density, comparing them to the state-of-the-art properties. Finally, the resulting advanced response contour plots from the DoE are discussed, a detailed discussion of which can be found in the thesis of Schelleis [5].

### 3.1. Fiber Migration and Fiber Length

**Figure 5:**
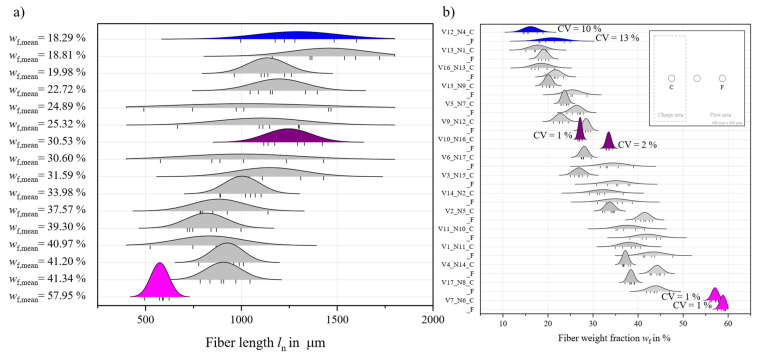
Ridgeline diagram showing the (**a**) distribution of *l*_n_ in relation to *w*_f_ (**b**) distribution of *w*_f_ in C and F for all factor combinations. Selected parameter combinations are highlighted [5].

The *w*_f_ is different between the C and F areas. Accordingly, the mechanical properties will be influenced by this fact. Results from TGA measurement are shown in Figure 5b, where a broad distribution of *w*_f_ between the C and F areas becomes apparent. The vertical lines at the bottom of each curve mark single measurements. Some measurements exhibit a low coefficient of variation (CV) marked by clear peaks and distinct differences between C and F, while in other cases, especially at low *w*_f_, the difference between the two areas is almost nonexistent. Generally, the CV decreases with *w*_f_.

The distributions of *l*_n_, shown in Figure 5a, do overlap a lot with almost no distinction between factor sets, especially at similar *w*_f_. Three factor sets are highlighted in color, corresponding to the factor settings resulting in lowest and highest *w*_f_ as well as one of the center points of the DoE at a medium *w*_f_. Measured *l*_n_ is in line with most current reported measurements (cf. Table 4). However, the measured *l*_n_ differs greatly from observations of the fiber skeleton of a burned-off plate, casting doubts on the validity of the measurement methods available. From just an optical assessment, fiber lengths exceed 50 mm. No conclusions about advantageous factors can be drawn from the data measured here. The use of fiber length as a quality feature is not recommended for LFT-D compression molding materials until more capable characterization methods are developed and validated.

### 3.2. Mechanical Properties

**Figure 6:**
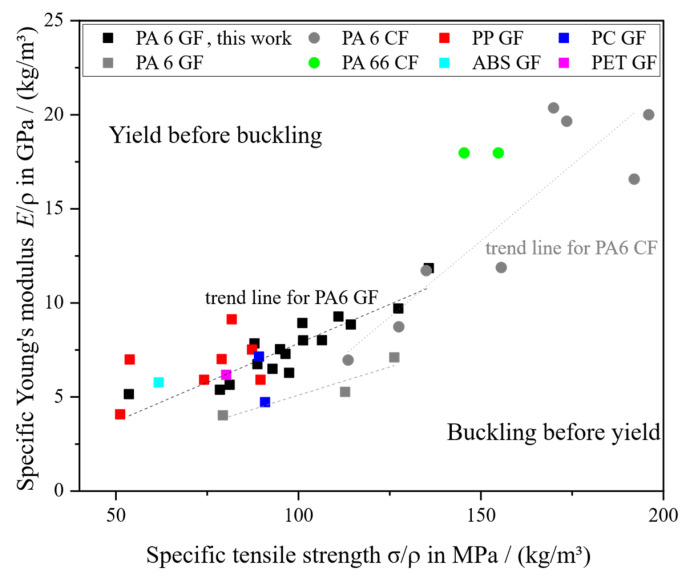
Specific tensile properties from this work in comparison to those presented in Table 5 and Table 6 [5].

**Figure 7:**
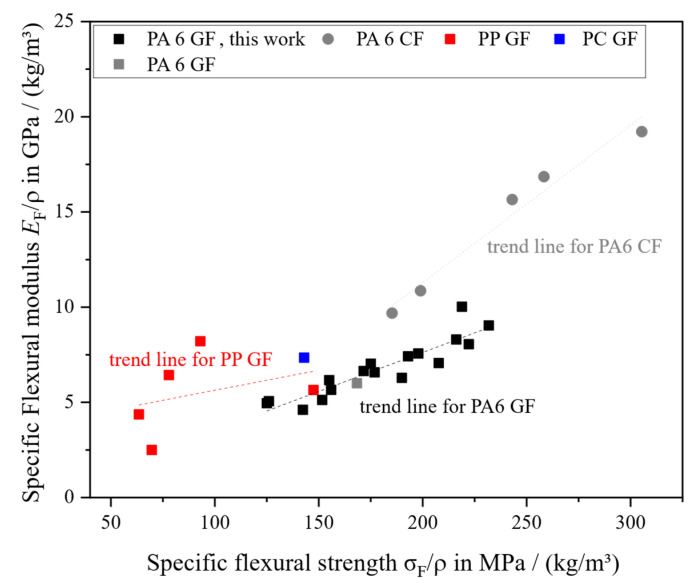
Specific flexural properties in comparison to those previously reported.

**Figure 8:**
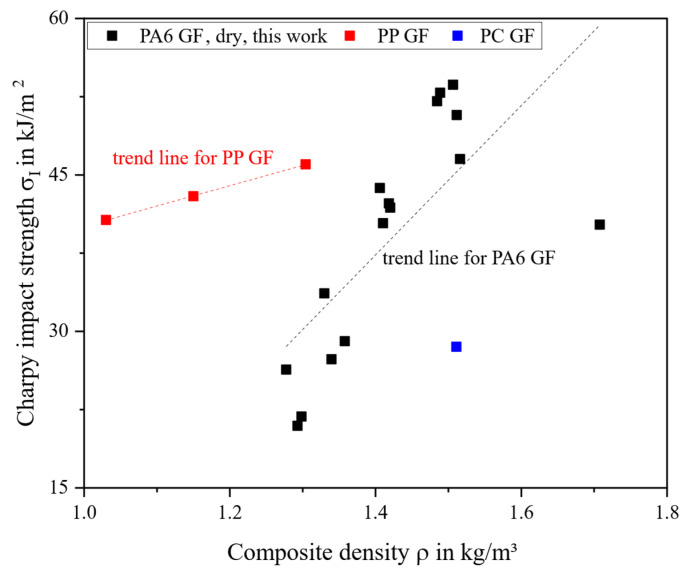
Charpy impact strength plotted over composite density in comparison to current literature.

Table 9 summarizes the mechanical properties and their respective standard deviations (std dev) from the study conducted here. Presented are samples from the F area in flow direction. They are sorted by ascending average *w*_f,F_ in the F area as it was determined by TGA and presented in Figure 5. The average *w*_f_ of the entire plate as well as the trial number are given for reference. The corresponding results for the C area are given in Table A1. These results have been partially discussed by Schelleis et al. [5,71,90]. Figure 6, Figure 7 and Figure 8, focus on classifying and comparing the results with the state-of-the-art results presented previously. All values are presented in relation to their respective composite density ρ, calculated according to reported or data sheet densities of polymer and fiber [17].

The tensile properties *E* and σ increase steadily with increasing *w*_f_; the Pearson coefficient is r = 0.99, indicating an almost perfect linear relationship. No levelling off of these curves towards high *w*_f_ can be observed. This is in contrast to the current research, where a plateau was observed for PP GF LFT-D [36] and PC GF LFT-D [24] at medium to high *w*_f_.

The coefficient of variation does increase from low *w*_f_, CV = 1%, to high *w*_f_, CV = 11%. This is in line with observations that mechanical properties in LFT-D materials are generally subject to significant fluctuations that originate from their heterogeneous microstructure. Figure 6 shows specific *E* plotted over specific σ in comparison to reported results (cf. Table 5 and Table 6). Not all reported results can be shown, as not all of them are documented in pairs of *E* and σ. A long corridor is available for selecting LFT-D materials. The CF reinforced materials clearly outperform most of the GF materials, however an area of overlap exists.

While tensile properties do not differ between the C and F areas considering *w*_f_, the flexural properties *E*_F_ and σ_F_ are distinctly different. This is due to the fiber orientation in the C area that is arranged in a shell-core structure similar to injection molding [57]. The F area in LFT-D compression molding does not have that structure [24,48,52]. In the C area the fibers in the outer layers, especially important to the bending load case, are oriented in the extrusion direction, usually perpendicular to the flow direction. For *E*_F_, the properties in the C area do not increase a lot with *w*_f_ like the corresponding samples from the F area. In the most extreme cases the C area samples are almost half that of corresponding samples in the F area.

The bending strength σ_F_ does have the same incline for the results of C and F areas like the tensile properties; however, both curves are offset. For both EF and σ_F_ in both areas, the linear relation with *w*_f_ is upheld with r between r = 0.91 and r = 0.97.

In Figure 7, the flexural properties are plotted with respect to their ρ. Much more distinct groupings of material combinations are visible, with PP GF LFT-D clearly demarcated towards the lower end and PA6 CF especially stiffer than PA6 GF while overlapping in strength at low CF and medium GF loadings. Comparable results for PA6 GF LFT-D were only published by Scheuring and fit well (grey square in Figure 7).

The impact strength is the only property tested decreasing towards the highest *w*_f_. The linear correlation is still high in the C area (r = 0.90) and F area (r = 0.86). Impact properties are susceptible to notches from the surface roughness [91]. This is especially true for the C area, where CV is high. In Figure 8, the dependence of PA6 on moisture becomes apparent; the dried specimens exhibit a brittle fracture mode and are no match for PP GF LFT-D materials, considering the performance-to-density ratio.

All previously shown plots exhibit a linear relation between the mechanical property and density, representative of *w*_f_. This is also apparent from recent contributions to the literature by Schelleis et al. [5,71,90].

### 3.3. Factor Influences on Mechanical Properties

Coefficient plots as well as response contour plots were generated from the DoE software after exclusion of insignificant model factors. Similar information across all output parameters can be obtained from coefficient plots. Increasing *n*_TSE_ and *n*_rov_ and decreasing *m*_p_ will lead to an improvement of the output parameters. Considering the connection between the factors and *w*_f_ shown in Figure 2, this is hardly surprising. All measures named will increase *w*_f_ and thus mechanical properties, as a linear relation was found. After coefficient normalization, the roving amount is the dominant factor regarding the output parameters. This is because the factor n_rov_ is tripled while the other factors *n*_TSE_ and *m*_pol_ are doubled. To obtain meaningful information, *w*_f_ must be considered. A response contour plot is a four-dimensional plot that displays the model’s predicted value for a chosen output parameter in relation to one factor while keeping the other factors fixed [92]. The axes of the plot can be chosen in a fashion matching those in Figure 2. Accordingly, process factor combinations can be located within the given parameter space.

**Figure 9:**
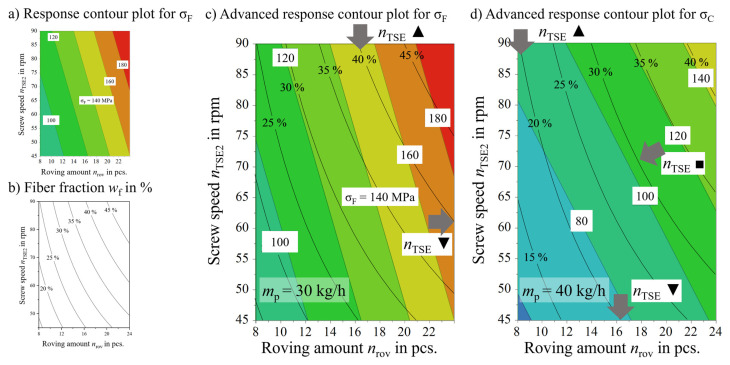
(**a**) Exemplary response contour plot for color-coded tensile strength σ. (**b**) Curves of constant *w*_f_ from Figure 2. (**c**,**d**) Combined plots from (**a**,**b**) into advanced response contour plot for process factor optimization, highlighting the choice between low ▼ and high ▲ *n*_TSE_ at comparable *w*_f_ (grey arrows). Based on [5].

Figure 9 shows an example of such a combination of Figure 9a response contour plot and Figure 9b *w*_f_ in the parameter space into an advanced response contour plot in Figure 9c. The lines of predicted tensile strength σ and *w*_f_ do largely but not completely match. From theory, σ should just increase with *w*_f_ until a saturation level, assuming a perfect fiber distribution. This linear increase is limited by fiber bundling and the accompanying decrease in the aspect ratio [13]. Following a line of constant *w*_f_ at a given *m*_p_ means that the total throughput *m*_LFT_ of TSE2 stays constant. The ratio $\dot{Q}$* is varied through *n*_TSE_ (cf. Equation (4)). With every change of *n*_TSE_, the roving amount *n*_rov_ must be adjusted to result in a constant *w*_f_. In practical application the *w*_f_ will be defined by the part design and the total throughput *m*_LFT_ will have to meet economic requirements. This leaves combinations of *n*_TSE_ and *n*_rov_ for optimization.

In Figure 9c, especially at higher *w*_f_, the choice of a low *n*_TSE_ (marked ▼, grey arrow on the right side of the plot) seems favorable when tracing the line of constant *w*_f_ (e.g., *w*_f_ = 40%), as σ is higher at predicted 170 MPa instead of 150 MPa at high *n*_TSE_ (marked ▲). Following the line at *w*_f_ = 25%, this clear choice of a low *n*_TSE_ is not so clear anymore; both ends of the line are in the light green area of predicted tensile strengths between 100 MPa and 120 MPa. This highlights the need to sort by *w*_f_ in addition to sorting the main factors. A discussion can take place based on this classification only.

Another plot is shown in Figure 9d, this is the advanced response contour plot for tensile strength in the C area σ_C_. Processing at the factor setting *m*_p_ = 40 kg/h and at low *w*_f_ = 20%, it is clear that a high *n*_TSE_ is the better choice. At medium *w*_f_ = 30%, the graph shows that changing *n*_TSE_ offers no potential for optimization (marked ■). Process factor recommendations can only be made based on individual mechanical properties and for specific microstructural regions C or F.

### 3.4. Recommended Parameter Settings

We have shown that the evaluations from the plots are very individual. Additionally, the transfer of conclusions to other material systems is only permissible after prior verification. The sensitivity to total LFT-D throughput governed by *m*_p_ and *w*_f_ is apparent from the plots so that an informed choice of *n*_TSE_ and *n*_rov_ can only be made under conditions as discussed before. For this purpose and following discussion, the parameter space was broken down according to *w*_f_ and *m*_p_ at low, medium and high levels. For every combination of *w*_f_ and *m*_p_, a recommendation regarding the choice of *n*_TSE_ can be made that is supported by the corresponding advanced response contour plot, as explained in Figure 9 and the accompanying discussion. Table 10 summarizes these recommendations for optimization in the F area by property. The factor setting of *n*_rov_ is thus determined by the choice of *w*_f_ and the other two factors.

Young’s modulus *E* has a clearly recognizable point where the factor recommendation turns at medium *w*_f_ and *m*_p_ (marked ■ in Table 10). For tensile strength σ in the F area the recommendation is very clear and processing at low *n*_TSE_ (marked ▼) is substantially better. Flexural modulus *E*_F_ is similarly clear but exactly the opposite. To improve *E*_F_ at any given *w*_f_, it should be processed at high *n*_TSE_ (marked ▲), highlighting the difficulties of an ideal factor choice for more than one output parameter. Flexural strength σ_F_ has a similar relation to *n*_TSE_ as *E*; the recommendation would be to process low *w*_f_ at low *n*_TSE_ and high *w*_f_ at high *n*_TSE_. For σ_F_, two exceptions exist that cannot be explained away by *m*_LFT_ or Q*. The impact toughness can be optimized by processing low *w*_f_ at low *n*_TSE_ and high *w*_f_ at high *n*_TSE_, with the tipping point at medium *m*_p_.

Weighing all recommendations for every *w*_f_ bracket individually, the conclusion is to process PA6 GF LFT-D with low *w*_f_ < 30% at low *n*_TSE_ and switch to high *n*_TSE_ for *w*_f_ > 40% for improved mechanical properties in the flow (F) area. A possible explanation for this switch is the growing importance of fiber de-bundling versus the preservation of *l*_f_ at higher *w*_f_ [30,39].

For 30% < *w*_f_ < 40%, the recommendation is unclear and exceptions for individual properties exist. It is also clear why contradictory statements from the current literature can all be true when other aspects of the respective experiments are not considered.

Processing recommendations for optimized properties in the C area are shown in Table 11. Because of the fiber migration [47] shifting *w*_f_ out of the C area, the high *w*_f_ bracket is not present for most properties and cannot be evaluated. Overall, in the C area there are more combinations of *w*_f_ and *m*_p_ where the impact of *n*_TSE_ is not clear (marked ■). This leaves room for other optimization criteria not discussed here. For example, the lofting of the plastificate is influenced by *n*_TSE_ and can be critical for handling. Influences of lofting on the fiber microstructure, especially fiber content [47] and orientation [51], were reported.

For the tensile properties *E* and σ, at all *w*_f_ and *m*_p_ levels a process recommendation must be issued that is contrary to the ones in the F area (Table 10). Flexural properties are influenced more by deviating fiber orientation, as the outer layers of samples are especially important [24,52]. Where *n*_TSE_ has optimization potential, it mostly aligns with the recommendations for the F area. The *n*_TSE_ recommendation for optimized impact toughness σ_I_ in the C area is similar to σ_I_ in the F area, as impact properties do not rely so much on fiber orientation [22]. Like the summarizing statement that can be deducted from Table 10, a picture emerges that is heterogeneous. Weighing the recommendations across all predicted mechanical properties in the C area, the conclusion is to process PA6 GF LFT-D with low *w*_f_ < 30% at low *n*_TSE_.

Due to the heterogeneous nature of the fiber microstructure of LFT-D materials characterized by changing *w*_f_, long fibers (cf. Figure 5) and fiber bundles, the CV of all mechanical properties is high. This can be a quality issue, as such deviations mean that correspondingly high safety factors must be applied in practice. The CVs are clustered by *w*_f_ and sorted by ascending *n*_TSE_ in these clusters. Linear fits of the resulting plots are drawn, and Pearsons R is evaluated. For the purposes of this discussion, it is assumed that at R > 0.5 (R < −0.5 respectively) there is a sufficient correlation between *n*_TSE_ in a *w*_f_ segment and CV to make a cautious factor recommendation supplementary to the preceding ones. The following Table 12 shows these recommendations sorted by *w*_f_ segments and divided by C and F area for all mechanical properties. Noticeably most correlations occur at high *w*_f_ > 40%. Between Table 11 and Table 12, hardly any consistent recommendations can be made.

## 4. Conclusions

This study presents the results of a structured approach to explore the process–microstructure–property relationships considering the close connection of processing factors and fiber content of LFT-D materials. An extensive list of reported LFT-D mechanical properties is given. A broad range of mechanical properties can be produced using different material combinations and seamlessly adjustable fiber contents. The complex main factor interactions of LFT-D compounding determining *w*_f_ are explained. Using the example of PA6 GF LFT-D, a DoE is run with the main factors, screw speed, fiber roving count and polymer throughput, resulting in a *w*_f_ range between *w*_f_ = 19% and *w*_f_ = 59%.

Advanced response contour plots are introduced and process recommendations for nTSE are derived and shown for tensile, flexural and impact properties. Those plots consider the intimate relation between the main factors and *w*_f_. Still, due to the complexity of factor interactions and microstructure development from compounding to compression molding, no simple recommendations can be made. For selected mechanical properties and their respective coefficients of variation in three *w*_f_ ranges, a recommendation regarding screw speed is given. The state-of-the-art approach, which is running TSE2 at low screw speeds, cannot be confirmed as the ideal solution and may be a bad choice for some output parameters. Optimizing selected output parameters requires an in-depth knowledge of the process–microstructure–property relation of LFT-D compression molding materials and needs to consider the continuous–discontinuous nature of the two processing phases. The plastificate is at the hinge between both compounding and compression molding processes. In this study, and in most literature, the compounding parameters are directly connected to the mechanical properties. There are intermediate variables at play—size and density of the plastificate, for example—that need to be investigated further to discuss the full picture of how the crucial fiber microstructure is formed.

## Figures and Tables

**Table 1:** Influence screw speed *n*_TSE_ in LFT-D compression molding on *l*_f_, *E* and σ [5].

*n*_TSE_ High/Low	Effect on	Summary	Source
Low ˅	*l*_f_ ˄	Less fiber attrition at reduced shear forces.	[25]
High ˄	*l*_n_, *l*_w_ ˅	More attrition decreases overall *l*_f_.	[14]
High ˄	*l*_f_ ˅	High fiber damage through high *n*_TSE_.	[22]
High ˄	*l*_n_ ˅	Total number of revolutions most significant factor for fiber fracture.	[32]
High ˄	*E*, σ ˄	Improved tensile and flexural properties. Better dispersion suspected.	[30]
High ˄	*E*, σ (˄)	Not significant but slightly higher tensile properties.	[39]
High/low ˄˅	*E*, σ >	Mixed results for screw speed variation. No significant effects found (PC GF LFT-D).	[24]
High ˄	*E*, σ ˄	Increasing tensile properties with *n*_TSE._	[40]

˄ and ˅ indicate a high or rising value, or a low or falling value. > indicates no effect on value.

**Table 2:** Influence of screw configuration in LFT-D compression molding on *l*_f_ as well as *E* and σ [5].

Shear High/Low	Effect on	Summary	Source
Low ˅	*l*_f_ ˅	Simple conveying elements also cause attrition of the fiber.	[22]
High ˄+	*a*_r_ ˄	Increase of chaotic flow will improve aspect ratio *a*_r_ through de-bundling.	[8,32]
High/Low ˄˅	*l*_f_ >	Little effect on *l*_f._	[32]
High ˄	FLD ˅	Increased shear will widen FLD.	[36]
High ˄	*l*_n_ ˅	Addition of GFM halves *l*_n_.	
High ˄	*E*, σ ˄	Addition of GFM improves tensile properties.	[42]
Low ˅	σ ˅	Poor impregnation and de-bundling of fibers cause low tensile strength.	[39]
High/low ˄˅	*E*, σ >	No influence of GFM on mechanical properties (PC GF LFT-D).	[41]

˄ and ˅ indicate a high or rising value, or a low or falling value. > indicates no effect on value.

**Table 3:** Influence of throughput *m*_LFT_ in general LFT extrusion processes [5].

*m*_LFT_ High/Low	Effect on	Summary	Source
High ˄	*t*_res_ ˅	Distribution of residence times tightens.	[38]
High ˄	*l*_w_ ˄	Number of longer fibers increases; stronger than influence of *n*_TSE_.	[14,37]
High ˄	*l*_f_ ˄	Probability of fiber damage is lower at higher *m*_LFT_.	[25]
High ˄	*l*_n_ ˄	Lower *t*_res_ leads to higher mean *l*_n_.	[43]
High ˄	*E*, σ >	Simultaneously increasing *m*_LFT_ cancels out effects of increased *n*_TSE_.	[25]

˄ and ˅ indicate a high or rising value, or a low or falling value. > indicates no effect on value.

**Table 4:** Reported fiber lengths *l*_f_ and *l*_n_ from LFT-D literature.

Material System	*w*_f_ in %	*l*_n_ in mm	*l*_w_ in mm	Source
PP GF	10–60	20	-	[36]
PP GF	25	11	-	[55]
PP GF	30	3	-	[56]
PP GF	30	1.2	15	[50]
PP GF	40	7	30	[62]
PA6 GF	30	1.5	5–10	[42]
PA6 GF	42	1.2	4.9	[53]
PA6 CF	9–25	0.3	-	[49]
PA6 CF	30–45	0.3	-	[30]
PA6 CF	33	6.4	1.6	[53]
PA6 CF	34	4.4	-	[63]
PC GF	20	0.8	-	[24]
PC GF	40	0.5	1.4	[40]

**Table 5:** Mechanical properties of glass-fiber-reinforced LFT-D materials.

Material	*w*_f_ in %	Young’s Modulus in GPa	Tensile Strength in MPa	Flex. Modulus in GPa	Flex. Strength in MPa	Impact Toughness in kJ/Nm^2^	
PP GF	10	3		2	67		[36]
	20	5					
	30	6					
	40	7					
	50	10	116	8	196		
	60	10	116	9			
PP GF	20	4 *					[69]
	30	7 *	100 *				
PP GF	30	8					[70]
PP GF	30		60				[68]
PP GF	40	8 *					[67]
PP GF	20	4	53	5	65	42	[71]
	34	7	85	7	90	49	
	48	12	107	11	121	60	
PA6 GF	30	10	173	8	230		[42]
PA6 GF	30	7 *	154 *				[72]
PA6 GF	30		112				[68]
PA6 GF	30	7	151				[65]
	45	11	175				
PA6 GF	41	13	189	11	284		[53]
PC GF	20	6	121				[24]
	40	11	130	11	216	43	
ABS GF	30	7 *					[67]
ABS GF	30		78				[68]
SAN GF	30	10 *					[67]
PET GF	30		126				[68]

* These values were extracted from a graph using image analysis.

**Table 6:** Mechanical properties of carbon-fiber-reinforced LFT-D materials.

Material	*w*_f_ in %	Young’s Modulus in GPa	Tensile Strength in MPa	Flex. Modulus in GPa	Flex. Strength in MPa	Impact Toughness in kJ/Nm^2^	
PA6 CF	9	8	134 *	7			[49]
	12	10	152 *	9			
	18	15	190 *	12			
	25	21	241 *	13			
PA6 CF	30	20 *	185 *	12 *	237 *		[30]
	35	20 *	184 *	14 *	260 *		
	40	24 *	196 *	21 *	325 *		
	45	26 *	198 *	23 *	353 *		
PA6 CF	33	26	254	25	396		[53]
PA66 CF	20		175				[39]
PA66 CF	40		255				[65]
PA66 CF	20	13	153				[73]
	30	18	178				
	35	24	190				
	40	24	185				

* These values were extracted from a graph using image analysis.

**Table 7:** Press closing profile comprising *w*_g_ and *v*_c_.

*w*_g_ in mm	*v*_c_ in mm/s
40	80
30	40
20	30
15	5
0	5

**Table 8:** Factor set points for *n*_TSE_, *m*_p_ and *n*_rov_ of the FCCD DoE with resulting *w*_f_.

*n*_TSE_ in rpm	*m*_p_ in kg/h	*n*_rov_ in pcs.	*w*_f_ in %
67.5	20	16	42.12
45	20	24	39.52
67.5	30	16	32.32
67.5	30	24	41.15
45	40	24	25.54
67.5	30	16	32.68
90	20	24	59.82
45	40	8	- *
67.5	40	16	26.06
67.5	30	16	32.17
90	30	16	38.65
90	40	8	19.97
45	20	8	19.72
90	20	8	33.63
45	30	16	23.26
67.5	30	8	19.84
90	40	24	41.32

* Not able to manufacture.

**Table 9:** Mechanical properties and standard deviation of 0° samples in the F area of PA6 GF LFT-D materials from this study sorted by ascending fiber weight content in the F area *w*_f,F_.

Material	*w*_f,F_ in %	*w*_f_ in %	Young’s Modulus (Std Dev) in GPa	Tensile Strength (Std Dev) in MPa	Flex. Modulus (Std Dev) in GPa	Flex. Strength (Std Dev) in MPa	Impact Toughness (Std Dev) in kJ/Nm^2^
PA6 GF	19.1	18.8	7.2 (0.4)	100 (6)	6.5 (0.6)	194 (32)	33.6 (6.1)
	20.9	18.3	6.6 (0.1)	73 (7)	6.5 (0.3)	163 (3)	27.1 (5.0)
	21.6	20.0	7.3 (0.4)	105 (5)	6.4 (0.1)	162 (10)	28.4 (2.3)
	25.3	22.7	8.6 (0.6)	123 (4)	7.5 (0.2)	207 (8)	44.7 (4.8)
	26.4	24.9	8.4 (0.8)	130 (16)	6.2 (0.3)	191 (7)	36.6 (4.1)
	28.4	25.3	9.1 (0.6)	120 (13)	8.4 (0.4)	211 (2)	39.5 (4.3)
	33.5	30.5	11.0 (0.8)	123 (6)	9.9 (0.6)	246 (9)	61.5 (9.2)
	33.9	30.6	11.2 (0.7)	142 (19)	9.3 (0.4)	250 (9)	56.9 (11.9)
	34.7	31.6	10.3 (0.8)	136 (8)	8.9 (0.1)	270 (31)	60.0 (11.7)
	34.9	33.5	10.6 (0.9)	134 (11)	9.4 (0.5)	244 (22)	59.5 (8.2)
	41.1	39.3	12.2 (1.4)	135 (21)	11.2 (0.6)	294 (15)	77.3 (13.7)
	41.5	37.6	13.3 (0.4)	164 (22)	10.5 (0.8)	309 (25)	78.7 (4.9)
	43.1	41.0	13.8 (1.3)	185 (16)	12.1 (1.0)	335 (28)	80.8 (9.7)
	43.9	41.3	13.3 (0.3)	178 (18)	12.6 (0.5)	328 (16)	70.5 (9.4)
	43.5	41.2	14.4 (0.9)	191 (7)	13.7 (0.5)	350 (24)	76.7 (5.9)
	58.9	58.0	20.0 (1.4)	229 (23)	17.1 (1.1)	374 (21)	68.7 (7.4)

**Table 10:** Process recommendations for individual mechanical properties in the F area regarding *n*_TSE_ settings depending on fiber fraction *w*_f_ and polymer throughput *m*_p_.

	*m*_p_ in kg/h	Tensile Properties		Flexural Properties		Impact Properties
		*E*	σ	*E*_F_	σ_F_	σ_I_
*w*_f_ = 20%	20	*n*_TSE_ ▼	*n*_TSE_ ▼	*n*_TSE_ ▲	*n*_TSE_ ▼	*n*_TSE_ ▼
-	30				*n*_TSE_ ▼	
*w*_f_ = 30%	40				*n*_TSE_ ▲	
*w*_f_ = 30%	20	*n*_TSE_ ▼	*n*_TSE_ ▼	*n*_TSE_ ▲	*n*_TSE_ ▼	*n*_TSE_ ▼
-	30	*n*_TSE_ ■			*n*_TSE_ ■	*n*_TSE_ ▲
*w*_f_ = 40%	40	*n*_TSE_ ▲		*n*_TSE_ ■	*n*_TSE_ ▲	*n*_TSE_ ▲
*w*_f_ = 40%	20	*n*_TSE_ ▲	*n*_TSE_ ▼	*n*_TSE_ ▲	*n*_TSE_ ▼	*n*_TSE_ ▲
-	30				*n*_TSE_ ▲	
*w*_f_ = 50%	40	-		*n*_TSE_ ■	*n*_TSE_ ▲	

*n*_TSE_ ▼ low *n*_TSE_ recommended; *n*_TSE_ ▲ high *n*_TSE_ recommended; *n*_TSE_ ■ choice of *n*_TSE_ not decisive.

**Table 11:** Process recommendations for individual mechanical properties in the C area regarding *n*_TSE_ settings depending on fiber fraction *w*_f_ and polymer throughput *m*_p_.

	*m*_p_ in kg/h	Tensile Properties		Flexural Properties		Impact Properties
		*E*	σ	*E*_F_	σ_F_	σ_I_
*w*_f_ = 20%	20	*n*_TSE_ ▲	*n*_TSE_ ▲	*n*_TSE_ ▲	*n*_TSE_ ■	*n*_TSE_ ▼
-	30					
*w*_f_ = 30%	40			*n*_TSE_ ■		
*w*_f_ = 30%	20	*n*_TSE_ ▲	*n*_TSE_ ▲	*n*_TSE_ ▲	*n*_TSE_ ▼	*n*_TSE_ ▼
-	30	*n*_TSE_ ■	*n*_TSE_ ■			*n*_TSE_ ■
*w*_f_ = 40%	40	*n*_TSE_ ▼		*n*_TSE_ ■		*n*_TSE_ ▲
*w*_f_ = 40%	20	*n*_TSE_ ▼	*n*_TSE_ ■	*n*_TSE_ ▲	*n*_TSE_ ▼	*n*_TSE_ ▲
-	30					
*w*_f_ = 50%	40	-	-	-	*-*	-

*n*_TSE_ ▼ low *n*_TSE_ recommended; *n*_TSE_ ▲ high *n*_TSE_ recommended; *n*_TSE_ ■ choice of *n*_TSE_ not decisive.

**Table 12:** Recommendations for *n*_TSE_ settings regarding low coefficient of variation CV in C and F areas in segments of *w*_f_.

	Sampling Area	Tensile Properties		Flexural Properties		Impact Properties
		*E*	σ	*E*_F_	σ_F_	σ_I_
*w*_f_ < 30%	C	-	-	*n*_TSE_ ▲ (−0.5)	-	-
	F	*n*_TSE_ ▲ (−0.8)	-	-	-	-
*w*_f_ > 30%	C	*n*_TSE_ ▲ (−0.5)	-	-	-	-
<40%	F	*n*_TSE_ ▼ (0.9)	-	-	-	*n*_TSE_ ▼ (0.5)
*w*_f_ > 40%	C		*n*_TSE_ ▼ (1.0)	*n*_TSE_ ▼ (1.0)	*n*_TSE_ ▼ (0.6)	
	F	*n*_TSE_ ▲ (−0.7)	*n*_TSE_ ▼ (0.7)	-	*n*_TSE_ ▲ (−0.9)	-

*n*_TSE_ ▼ low *n*_TSE_ recommended; *n*_TSE_ ▲ high *n*_TSE_ recommended.

## Data Availability

The raw data supporting the conclusions of this article will be made available by the authors on request.

## Abbreviations

The following abbreviations are used in this manuscript:

ABS	Acrylonitrile butadiene styrene
C	Charge area
CF	Carbon fiber
Co	Continuous (FRP)
CV	Coefficient of variation
DiCo	Discontinuous (FRP)
DoE	Design of experiments
F	Flow area
FLD	Fiber length distribution
FOD	Fiber orientation distribution
FRP	Fiber reinforced polymer
GF	Glass fiber
GFM	Type of TSE screw mixing element
GMT	Glass mat thermoplast
LFT	Long-fiber thermoplastic
LFT-D	LFT direct compounding
PA	Polyamide
PC	Polycarbonate
PET	Polyethylenterephthalat
PP	Polypropylene
SAN	Styrene acrylonitrile resin
SMC	Sheet molding compound
SME	Specific mechanical energy
TSE	Twin screw extruder

## Appendix A

**Table A1:** Mechanical properties and standard deviations of 0° samples in the C area of PA6 GF LFT-D materials from this study.

Material	*w*_f,C_ in %	*w*_f_ in %	Young’s Modulus (Std Dev) in GPa	Tensile Strength (Std Dev) in MPa	Flex. Modulus (Std Dev) in GPa	Flex. Strength (Std Dev) in MPa	Impact Toughness (Std Dev) in kJ/Nm^2^
PA6 GF	16.1	18.3	6.8 (0.3)	102 (4)	4.7 (0.2)	101 (10)	20.4 (4.5)
	17.7	18.8	6.8 (0.4)	72 (2)	4.6 (0.4)	102 (17)	24.4 (6.3)
	18.5	20.0	6.9 (0.5)	75 (5)	4.6 (0.2)	107 (4)	23.0 (6.2)
	20.1	22.7	7.1 (0.4)	99 (8)	4.5 (0.2)	134 (4)	29.0 (7.1)
	22.8	25.3	8.0 (0.4)	87 (3)	5.4 (0.2)	124 (9)	31.5 (2.7)
	23.7	24.9	7.9 (0.1)	98 (4)	4.6 (0.0)	138 (3)	28.3 (2.9)
	26.9	31.6	8.9 (0.6)	103 (4)	5.5 (0.1)	141 (1)	44.3 (6.8)
	27.1	30.5	9.9 (0.9)	104 (7)	5.7 (0.2)	118 (3)	47.6 (6.4)
	28.0	30.6	9.6 (0.9)	100 (13)	5.1 (0.4)	119 (11)	41.3 (9.7)
	32.2	33.5	9.0 (0.2)	99 (11)	6.0 (0.2)	134 (7)	37.5 (8.7)
	33.7	37.6	10.4 (0.7)	130 (8)	4.9 (0.2)	141 (9)	54.9 (10.5)
	37.2	41.2	12.3 (0.6)	149 (12)	6.4 (0.3)	169 (6)	55.5 (10.8)
	37.6	39.3	11.2 (0.7)	143 (3)	6.4 (0.1)	150 (7)	52.7 (5.5)
	38.0	41.0	12.1 (1.0)	143 (13)	7.1 (0.6)	190 (10)	57.9 (4.6)
	38.4	41.3	12.2 (0.4)	144 (5)	6.4 (0.2)	163 (2)	54.4 (12.2)
	57.1	58.0	17.0 (1.7)	198 (6)	11.2 (1.1)	266 (9)	62.1 (26.7)
